# Incidence and antibiotic prescribing for clinically diagnosed urinary tract infection in older adults in UK primary care, 2004-2014

**DOI:** 10.1371/journal.pone.0190521

**Published:** 2018-01-05

**Authors:** Haroon Ahmed, Daniel Farewell, Hywel M. Jones, Nick A. Francis, Shantini Paranjothy, Christopher C. Butler

**Affiliations:** 1 Division of Population Medicine, Cardiff University School of Medicine, Cardiff, United Kingdom; 2 Nuffield Department of Primary Care Health Sciences, University of Oxford, Oxford, United Kingdom; Universidade Nova de Lisboa Instituto de Higiene e Medicina Tropical, PORTUGAL

## Abstract

Urinary tract infections (UTI) are an important cause of morbidity and antibiotic use in older adults but there are little data describing disease burden in primary care. The aim of this study was to estimate the incidence of clinically diagnosed UTI and examine associated empirical antibiotic prescribing. We conducted a retrospective observational study using linked health records from almost one million patients aged ≥65 years old, registered with 393 primary care practices in England. We estimated incidence of clinically diagnosed UTI between March 2004 and April 2014, and used multilevel logistic regression to examine trends in empiric antibiotic prescribing. Of 931,945 older adults, 196,358 (21%) had at least one clinically diagnosed UTI over the study period. In men, the incidence of clinically diagnosed UTI per 100 person-years at risk increased from 2.81 to 3.05 in those aged 65–74, 5.90 to 6.13 in those aged 75–84, and 8.08 to 10.54 in those aged 85+. In women, incidence increased from 9.03 to 10.96 in those aged 65–74, 11.35 to 14.34 in those aged 75–84, and 14.65 to 19.80 in those aged 85+. Prescribing of broad-spectrum antibiotics decreased over the study period. There were increases in the proportion of older men (from 45% to 74%) and women (from 55% to 82%) with UTI, prescribed a UTI specific antibiotic. There were also increases in the proportion of older men (42% to 69%) and women (15% to 26%) prescribed antibiotics for durations recommended by clinical guidelines. This is the first population-based study describing the burden of UTI in UK primary care. Our findings suggest a need to better understand reasons for increasing rates of clinically diagnosed UTI and consider how best to address this important clinical problem.

## Introduction

Urinary tract infection (UTI) is an important cause of morbidity and antibiotic use in older adults. Estimates suggest community dwelling older adults experience around 11 episodes per 100 years at risk. [1] Incidence is higher in older adults with diabetes, [2] and in the very frail.[3] In three studies of care home residents, UTI accounted for 29%,[4] 47% [5] and 66% [6] of all antibiotic prescriptions. There is increasing evidence that hospital admission for UTI is increasing in the UK [7] and the US [8] and thus UTI is becoming an important cause of health service use in older adults.

Antibiotic prescribing for UTI is associated with an increased risk of antibiotic resistant urinary pathogens that persist for at least twelve months after prescription. [9] Antibiotic stewardship strategies advocate using narrow spectrum antibiotics for the shortest duration required for clinical recovery. UK clinical guidelines recommend nitrofurantoin or trimethoprim as first-line antibiotic therapy for UTI.[10] Guidelines, supported by data from meta-analyses of randomised trials,[11] advise three days of antibiotic therapy for UTI in adult women. For men, there is a lack of empirical research comparing different antibiotic durations, but expert consensus recommends seven days of antibiotic therapy.[10]

In the UK, most episodes of suspected UTI are manged in primary care. Despite the associated morbidity, there are few recent, robust, externally valid data describing trends in the incidence of UTI in UK primary care. A large prospective observational study with systematic urine sampling would provide estimates of incidence of microbiologically confirmed UTI, but would be expensive and pose several challenges including recruitment, retention and collection of uncontaminated urine samples. It would also not reflect the true burden of UTI in primary care as many episodes are diagnosed and treated clinically, based on symptoms and signs, without microbiological confirmation. Therefore, we used anonymised linked health record data from nearly one million older adults to estimate incidence rates of clinically diagnosed UTI in UK primary care and examine associated antibiotic prescribing. We investigated trends in the proportion of UTIs prescribed nitrofurantoin and trimethoprim, and the proportion prescribed antibiotics for durations recommended in clinical guidelines.

## Methods

### Data source

We conducted a retrospective observational study using the Clinical Practice Research Datalink (CPRD). The CPRD is an electronic database of routinely collected primary care data, covering 11.3 million patients from 674 general practices across the UK.[12] Approximately 7% of the UK population are included and patients are broadly representative of the wider UK population in terms of age, sex and ethnicity. The CPRD holds anonymised data on demographics, drug prescriptions, laboratory tests, specialist referrals, and clinical diagnoses. Clinical signs, symptoms and diagnoses are recorded using the hierarchical Read code system, which has been used across almost all UK primary care practices since the mid-1990s.[13] CPRD data are available once they have met a series of quality checks on completeness and reliability and deemed to be of a required standard for research purposes. Linked hospital data is available for patients from approximately 50% of contributing English practices. Hospital diagnoses are recorded using version 10 of the International Classification of Disease (ICD-10). Linked hospital data were required for two reasons. Firstly, although most hospital admission related diagnoses are recorded in primary care records, previous research has shown linked hospital admission data improves case detection.[14] Secondly, patients would not be at risk of a community acquired UTI during a period of hospitalisation and thus, this time would need to be subtracted from the time-at-risk. Hospital records provide more reliable records of admission and discharge dates.

### Population

Patients were eligible for inclusion if they were ≥65 years old, had linked hospital data and more than one day of CPRD follow-up between 1^st^ March 2004 and 31^st^ March 2014. We excluded patients with temporary registrations or gaps in their data coverage. Patient follow-up began on the latest of study start date, the patient’s 65^th^ birthday or 28 weeks after the patient first registered at the practice to avoid including historical illnesses recorded at registration. Follow-up ended at the earliest of study end date, death, or last day of available CPRD data.

### Case ascertainment

To maximise chances of identifying episodes of UTI and reduce chances of identifying asymptomatic bacteriuria, we identified cases as follows. **All** potential cases **must** have had a record of a primary care consultation with Read codes indicating either a diagnosis of UTI or a clearly relevant symptom of UTI, for example, dysuria or urinary frequency. They then needed at least one of the following:, 1) a same-day antibiotic prescription (suggesting a primary care clinically diagnosed and empirically treated UTI), 2) a same-day emergency hospital admission with an ICD-10 code for UTI (suggesting a primary care clinically diagnosed UTI confirmed in secondary care), or 3) a same-day Read code indicating urine was sent for culture, *and* an antibiotic prescription within seven days (suggesting a primary care clinically suspected UTI, confirmed and treated following culture). The flowchart and Read and ICD-10 codes used for case ascertainment are available in the supporting information (S1 File).

To account for multiple consultations for the same illness episode, we considered UTI related codes within 28 days of one another to belong to the same episode. We chose 28 days to be consistent with previous linked health record research on infection incidence, [2, 15] and recognised this may lead to conservative estimates. To ensure we only included incident *community acquired* UTI, we considered UTI episodes within 14 days of a hospital discharge (identified from linked hospital data) to be hospital acquired and therefore excluded these from the numerator.

### Statistical analyses

We calculated age and gender specific incidence rates and 95% confidence intervals (CIs) for each month from March 2004 to April 2014 by dividing the number of incident UTIs by person-time at risk. Individuals were considered not at risk of an incident community acquired UTI if they were in hospital, for 14 days following a hospital discharge, and for periods of time following an incident UTI until they had 28 days without a UTI related code. We multiplied calculated incident rates by 365 X 100 to transform from incidence per person-days at risk to incidence per 100 person-years at risk. Incidence rates were calculated for three age groups: 65–74, 75–84 and 85+ years. We calculated the mean age within each age group for each study year to assess if any changes in incidence rate were due to increasing age within that group.

We used joinpoint regression to model trends in incidence rates over time and identify the estimated location of any significant change in the slope of a trend line.[16] Joinpoint analysis identifies the best fit for inflexion points (“joinpoints”) at which there is a significant change in trends using a series of permutation tests. [17] In this study, joinpoint analysis was used to identify months (as the independent variable) at which significant changes in incidence rates occurred over the study period, and the size of these changes (as the percentage change in rate per year). A maximum of two joinpoints were allowed for each model we considered. This was the default value according to the number of observations in each model. We estimated the annual percentage change and 95% confidence intervals for each trend line.

For the group of individuals prescribed a same-day empirical antibiotic in primary care, we investigated gender-specific trends for antibiotic choice and duration. We used multilevel logistic regression to account for clustering within practices and modelled trends in (i) the proportion of older adults prescribed a UTI specific antibiotic (trimethoprim or nitrofurantoin), and (ii) the proportion of older adults prescribed antibiotics for the duration recommended by clinical guidelines (three days for women, seven days for men).

Analyses were undertaken in R version 3.3.1. and Joinpoint Regression Program version 4.3.1.0. The CPRD Independent Scientific Advisory Committee approved the study protocol (protocol number 17_098). Further ethical approval was not required as the proposed research was within the remit of the CPRDs broad National Research Ethics Service approval. We used the Reporting of Studies Conducted using Observational Routinely-collected Health Data (RECORD) statement and checklist to guide study reporting.[18]

## Results

There were 966,454 adults aged ≥65 with data of acceptable standard, linked hospital data, and at least one day of follow-up between 2004 and 2014, in the database. We excluded 34,509 (3.6%), resulting in a final study population of 931,945 older adults (S1 Fig). Table 1 shows study population characteristics.

**Table 1:** Characteristics of the study population.

	Number (%)
Total study population	931945
Male	417190 (45)
Female	514755 (55)
Median (IQR) age at start of follow-up (years)	70.2 (65.0–78.2)
Median (IQR) age at end of follow-up (years)	77.1 (70.3–84.4)
Median (IQR) follow-up time (years)	5.0 (2.2–8.5)
Total follow-up time (person years)	4,857,433

### Incidence by age and gender

Of 931,945 older adults, 196,358 (21%) had at least one UTI between 1^st^ March 2004 and 31^st^ March 2014. In this cohort of 196,358 patients, we identified 450,080 episodes of community acquired UTI. Median number of episodes per person was 2 (IQR 1–4). Over 96% of episodes were identified by the presence of a diagnostic (e.g., “Urinary tract infection”) or symptomatic (e.g., “dysuria”) Read code and a same-day antibiotic prescription. Incidence of UTI increased with age and was higher in women. There was marked monthly variation in incidence for both men and women, but with no clear pattern or seasonal distribution.

**Fig 1:**
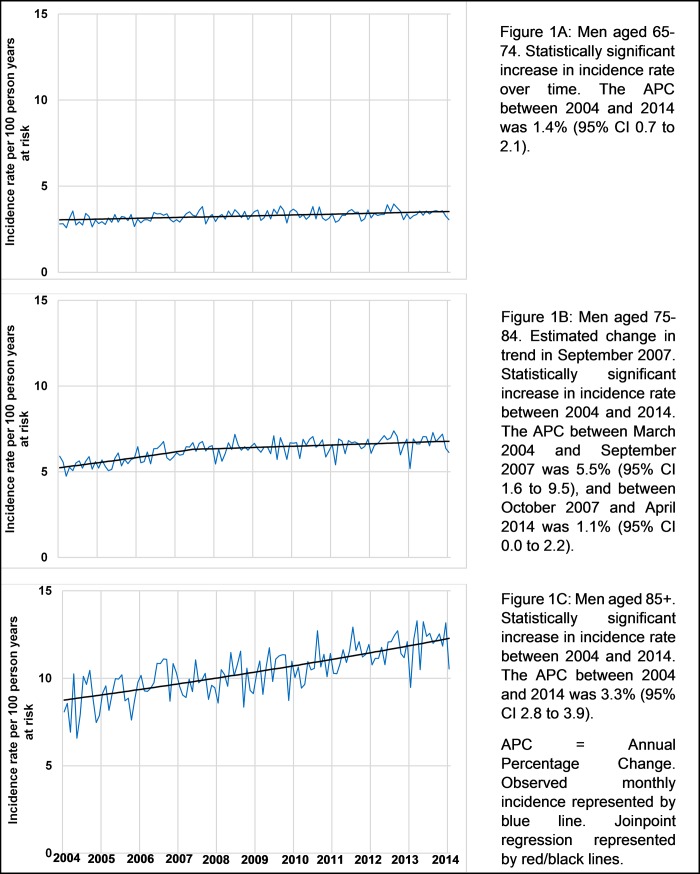
Joinpoint analyses of monthly age-specific community acquired urinary tract infection incidence rates for older men in UK primary care March 2004 –April 2014.

The incidence of UTI in older men (episodes per 100 person-years at risk), increased between March 2004 and April 2014 from 2.81 (95% CI, 2.48–3.15) to 3.05 (95% CI, 2.56–3.54) in those aged 65–74, and 5.90 (95% CI, 5.28–6.53) to 6.13 (95% CI, 5.25–7.00) in those aged 75–84 (S1 Table). Increase was most marked in those aged 85+, from 8.08 (95% CI, 6.64–9.52) to 10.54 (95% CI, 8.61–12.48). Mean age within each age group was stable over the study period (S2 Table). Joinpoint analyses showed an annual percentage increase (APC) in incidence rates of 1.4% (95% CI, 0.7–2.1) in those aged 65–74 (Fig 1). The APC for those aged 75–84 was 5.5% (95% CI, 1.6–9.5) between March 2004 and September 2007, followed by a change in trend in September 2007 (95% CI, May 2006 to January 2009), and then an APC of 1.1% (95% CI, 0.0–2.2) between October 2007 and April 2014. The APC for those aged 85+ was 3.3% (95% CI, 2.8–3.9).

**Fig 2:**
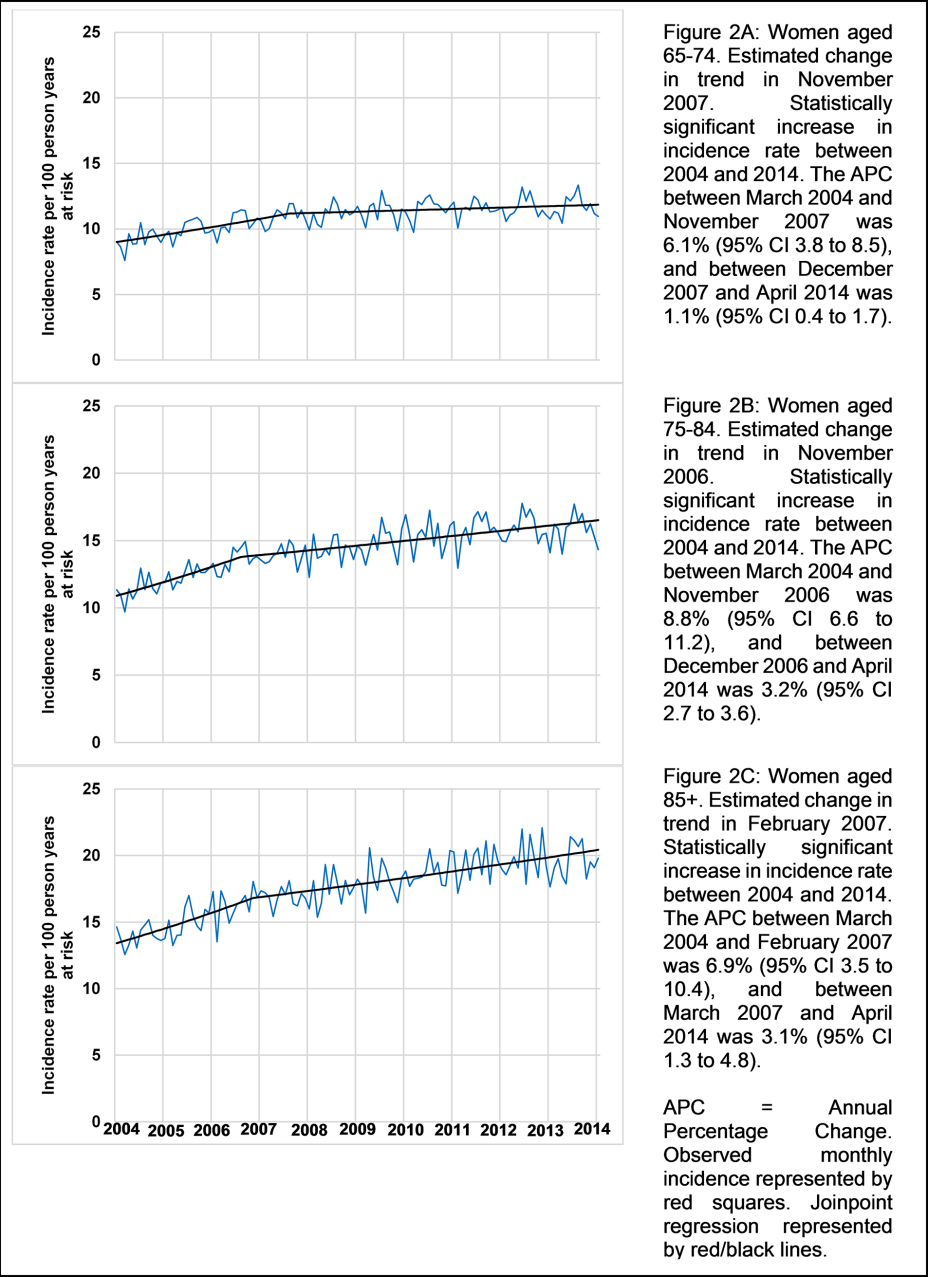
Joinpoint analyses of monthly age-specific community acquired urinary tract infection incidence rates for older women in UK primary care March 2004 –April 2014.

The incidence of UTI in older women (episodes per 100 person-years at risk), increased between March 2004 and April 2014 from 9.03 (95% CI, 8.44–9.61) to 10.96 (95% CI, 10.05–11.87) in those aged 65–74, 11.35 (95% CI, 10.62–12.07) to 14.34 (95% CI, 13.13–15.54) in those aged 75–84, and 14.65 (95% CI, 13.39–15.91) to 19.80 (95% CI, 17.86–21.73) in those aged 85+ (S1 Table). Mean age within each age group was stable over the study period (S2 Table). The APC for those aged 65–74 was 6.1% (95% CI, 3.8 to 8.5) between March 2004 and November 2007, and 1.1% (95% CI, 0.4 to 1.7) between December 2007 and April 2014 (Fig 2). The APC for those aged 75–84 was 8.8% (95% CI, 6.6 to 11.2) between March 2004 and November 2006, and 3.2% (95% CI, 2.7 to 3.6) between December 2006 and April 2014. The APC for those aged 85+ was 6.9% (95% CI, 3.5 to 10.4) between March 2004 and February 2007, and 3.1% (95% CI, 1.3 to 4.8) between March 2007 and April 2014. Estimated changes in trend for the 65–74, 75–84 and 85+ age groups occurred in December 2007 (95% CI, May 2006 to April 2009), November 2006 (95% CI, February 2006 to January 2008), and February 2007 (95% CI, January 2006 to April 2009).

### Antibiotic choice

**Fig 3:**
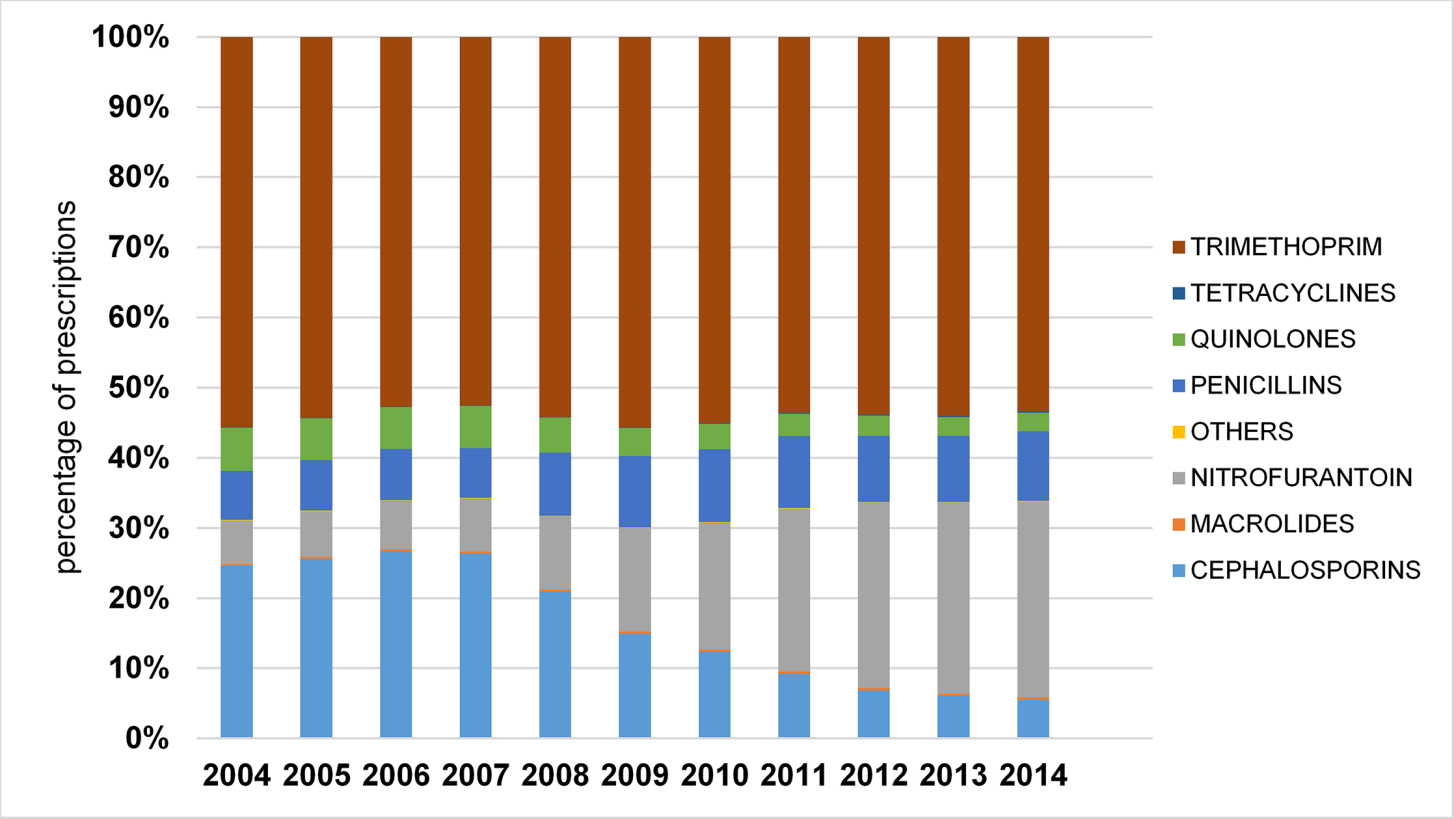
Antibiotic prescribing for community acquired UTI for older women by year and antibiotic group.

**Fig 4:**
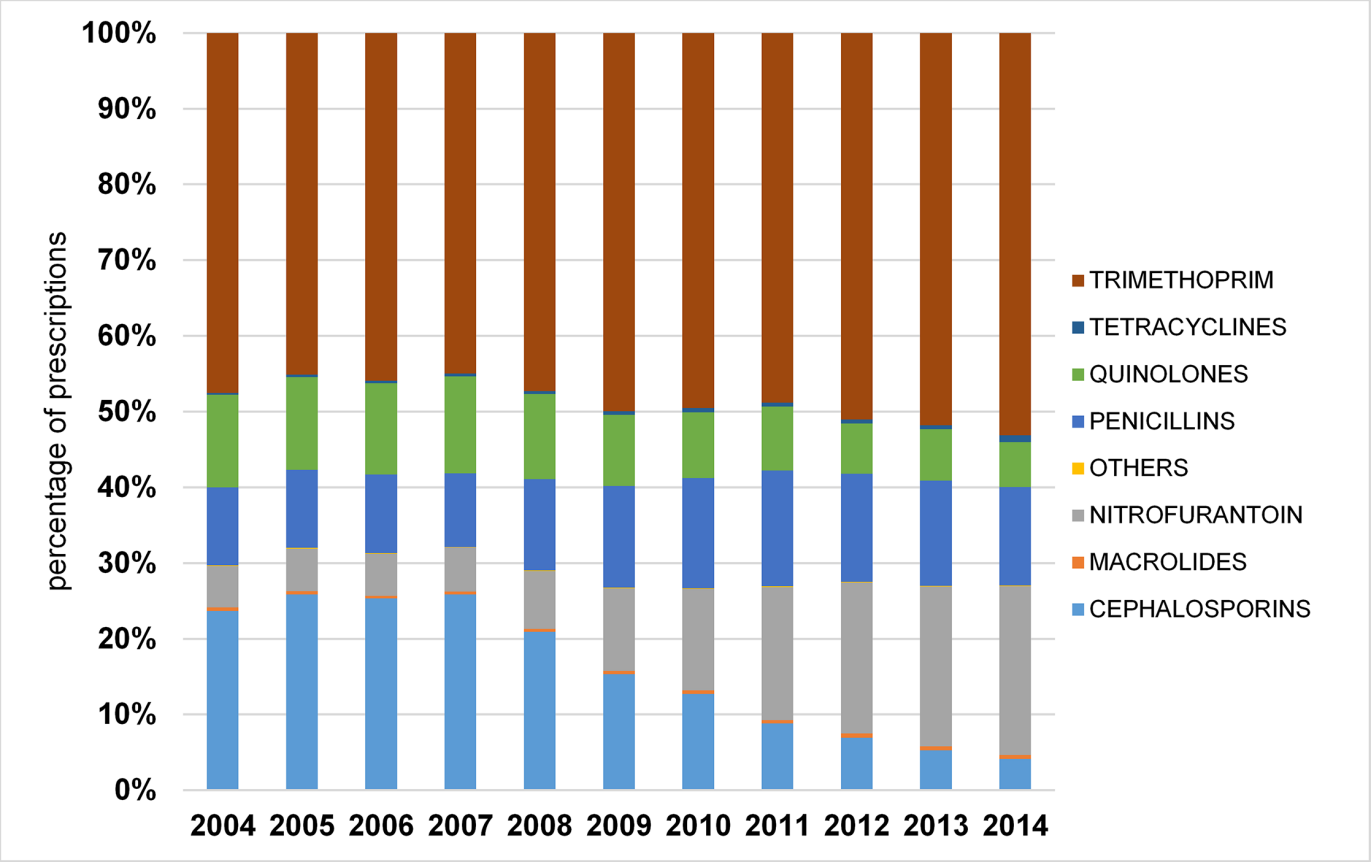
Antibiotic prescribing for community acquired UTI for older men by year and antibiotic group.

Trends in antibiotic choice were similar for older men and women (Figs 3 and 4). Trimethoprim was consistently the most commonly prescribed antibiotic for community acquired UTI, accounting for about 50% of all prescriptions. Prescriptions of broad-spectrum cephalosporins for UTI decreased markedly in men from 23.7% in 2004 to 4.1% in 2014, and women from 24.6% in 2004 to 5.5% in 2014. Quinolone use also decreased; in men from 12.2% in 2004 to 6% in 2014 and in women, from 6.2% in 2004 to 2.7% in 2014. Prescriptions of nitrofurantoin for community acquired UTI increased markedly during the study period, rising from 5.5% of prescriptions for male UTI in 2004, to 22.3% in 2014, and from 6.2% of prescriptions for female UTI in 2004 to 27.9% in 2014. Use of other antibiotic groups remained relatively stable.

Multi-level logistic regression showed an increase in the proportion of older men prescribed a UTI specific antibiotic (nitrofurantoin or trimethoprim) between March 2004 and April 2014, from 45% to 74%. The parameter estimates suggest that a practice with UTI specific prescribing one standard deviation below the mean would show an increase across the 10-year study period from 24% to 75%, and a practice with prescribing one standard deviation above the mean would show an increase from 67% to 95%. Across the same period, there was also an increase in the proportion of older women prescribed a UTI specific antibiotic, from 55% to 82%. Model estimates suggest that a practice with UTI specific antibiotic prescribing one standard deviation below the mean would show an increase from 31% to 85% and a practice with prescribing one standard deviation above the mean would show an increase from 76% to 97%.

### Duration of antibiotic prescription

Multi-level logistic regression showed an increase in the proportion of older men prescribed seven-day antibiotic therapy between 2004 and 2014, from 42% to 69%. Model estimates suggest a practice with seven-day therapy prescribing one standard deviation below the mean would show an increase from 23% to 74%, and a practice with seven-day therapy prescribing one standard deviation above the mean would show a change from 64% to 94%. Across the same period, there was also an increase in the proportion of older women prescribed three day antibiotic therapy, from 15% to 26%. Model estimates suggest that a practice with three-day therapy prescribing one standard deviation below the mean would show a change from 4% to 31% and a practice with three-day therapy prescribing one standard deviation above the mean would show a change from 43% to 90%.

## Discussion

### Summary

This study is the first to provide age and gender-specific monthly incidence estimates of clinically diagnosed UTI derived from a large population-based sample. We identified monthly variation in incidence rates with an overall increasing incidence rate that was most marked for men over 85 and women over 75. About 20% of nearly one million older adults in our sample had at least one clinically diagnosed UTI in primary care over a 10-year period. The proportion of older adults prescribed nitrofurantoin or trimethoprim increased, as did the proportion of older men prescribed seven-day antibiotic therapy. However, only one in four older women were prescribed three-day therapy as recommended by guidelines, suggesting on-going clinical uncertainty in this area.

### Strengths and limitations

Our study used a large population-based sample to estimate UTI incidence trends. We sought to optimise the accuracy of our estimates by calculating days at risk for included individuals, and subtracted time at risk of hospital acquired infection from the denominator. We distinguished repeat consultations for the same infection from new incident infection by attributing codes within 28 days of one another to the same episode. This may have underestimated the incidence of UTI if some of these episodes were actually new incident UTIs. We did not have access to linked microbiological data and thus the UTI episodes are clinically diagnosed episodes rather than microbiologically confirmed, but as over 98% of these episodes were associated with a same-day antibiotic prescription, they are more likely to reflect the true burden of clinically diagnosed and empirically treated UTI in a primary care population. However, data used were recorded for clinical purposes and thus are prone to a degree of coding error, differential coding between clinicians, and confounding by indication. We also would not have captured incident UTIs where the antibiotic prescription was associated with a non-specific code (e.g., “patient reviewed”).

### Comparison with existing literature

Our incidence estimates are broadly consistent with previous population-based studies. In the Leiden 85-plus study, the incidence of physician diagnosed UTI over 1246 person years at risk for those aged 86 through 90 years was 11.2 episodes per 100 person-years at risk.[19] In contrast to two previous studies, [20, 21] we did not identify any clear evidence of seasonality. We did identify a change in incidence trend for older women occurring around 2007, with a reduction in the APC for incidence rates that followed this period. Reasons for this could include new guidelines published in 2006, [22] increased publicity around antimicrobial stewardship, or a shift towards more criteria based diagnosis of UTI in older people, which has been associated with reduced antibiotic use for UTI.[23, 24] Our analyses demonstrated increasing incidence of UTI, especially in men over 85 and women over 75. This may represent over-diagnosis, reflecting the increasing challenge of accurately diagnosing UTI in this population, or may represent an increase in true bacterial UTI, possibly due to the increasing prevalence of elderly multi-morbid individuals with greater susceptibility to infections. The lack of microbiological data prevents further exploration of possible causes with CPRD data, as were are unable to ascertain who had a true microbiologically confirmed UTI. Further investigation with alternative data-sources and methods is warranted to ascertain the reasons for this increase and to assess whether preventative or diagnostic interventions could effectively and safely reduce incidence and associated antibiotic use.

Guideline congruent antibiotic prescribing for community acquired UTI is improving, with increasing use of UTI specific antibiotics, a reduction in broad-spectrum antibiotic prescribing, and greater adherence to prescribing of seven-day antibiotic therapy for men. Empirical evidence for optimal antibiotic duration in older men is limited. A claims registry based US observational study showed no difference in clinically important outcomes between men with UTI prescribed <7 days of antibiotics versus those prescribed 7 days or more.[25] However, in elderly women there is empirical trial evidence for optimal antibiotic duration for UTI, with meta-analysis showing no difference in short (3 trials, N = 431) or long-term (3 trials, N = 470) outcomes between those treated with three days of antibiotics versus those treated with seven days.[11] Despite this, prescribing antibiotic therapy for three days for older women remained relatively uncommon, with almost 75% still receiving a prescription for longer. Previous studies have found that clinician adherence to evidence based guidance for UTI is sub-optimal,[26] in part due to conflicting recommendations in guidelines,[27] and clinical complexity and prognostic uncertainty associated with UTI in older adults.[28, 29] There may be potential for improving management of UTI in older women through better understanding the uncertainties around recovery and prognosis from short versus long courses of antibiotics.

## Conclusions

This population-based analysis of clinical records from nearly one million older adults has shown an increase in the incidence of clinically diagnosed UTI between 2004 and 2014. There is a clear need to better understand the reasons for the increasing incidence, and for interventions that improve prevention and diagnosis of UTI. Although antibiotic choice for UTI in primary care has improved, further improvements could arise through better understanding and addressing the reasons for the relatively low uptake of short-course therapy for older women.

## Supporting information

S1 Fig Study flow diagram. (DOCX)

S1 File Identifying clinically diagnosed UTI using Read and ICD-10 codes. (DOCX)

S1 Table Incidence rates per 100 person years at risk for community acquired UTI 2004–2014. Every third month shown.
(DOCX)

S2 Table Mean age (years) for each age group for each study year. (DOCX)
