# Supplementary figures and images for: Incidence and antibiotic prescribing for clinically diagnosed urinary tract infection in older adults in UK primary care, 2004-2014
Source: PLoS One. 2018 Jan 5;13(1):e0190521. doi: 10.1371/journal.pone.0190521 (PMC5755802; doi:10.1371/journal.pone.0190521)

**S1 Fig: Study flow diagram**


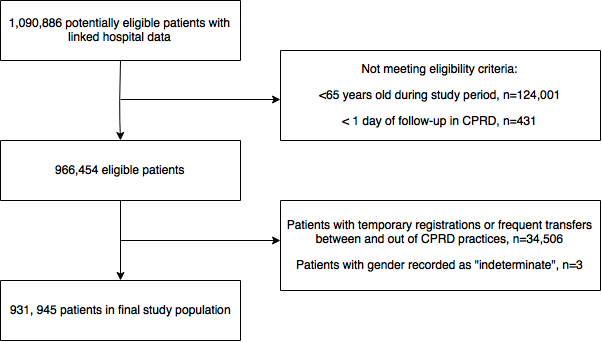

Supplement: S1 Fig — (DOCX) [file pone.0190521.s001.docx]
