# Supplementary material for: Incidence and antibiotic prescribing for clinically diagnosed urinary tract infection in older adults in UK primary care, 2004-2014
Source: PLoS One. 2018 Jan 5;13(1):e0190521. doi: 10.1371/journal.pone.0190521 (PMC5755802; doi:10.1371/journal.pone.0190521)
Supplement: S1 File — (DOCX) [file pone.0190521.s002.docx]

**S1 File: Identifying clinically diagnosed UTI using Read and ICD-10 codes**


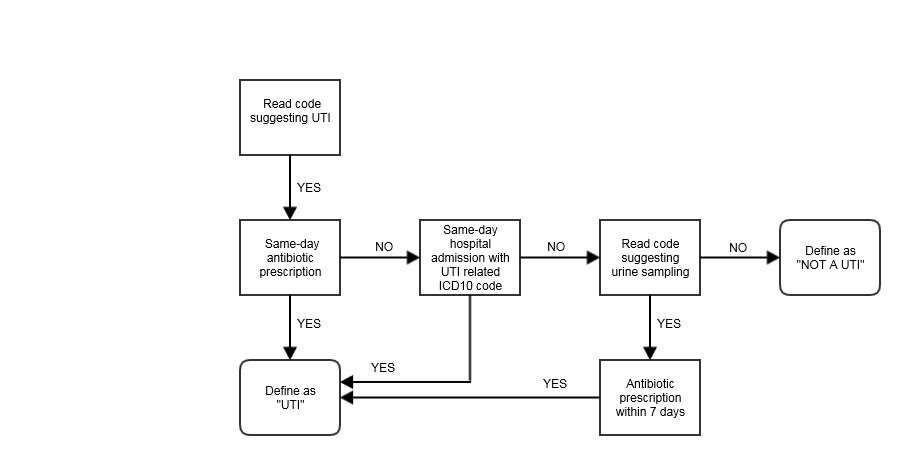


NO

See code list 2

See code list 3

See code list 1

**Code List 1: Read codes suggesting UTI**

**All cases were required to have one of the following codes indicating a diagnosis of UTI or a clinically relevant symptom.**

| \| **Read code** \| **readterm** \| \| --- \| --- \| \| K190300 \| Recurrent urinary tract infection \| \| K190.11 \| Recurrent urinary tract infection \| \| 1AG..00 \| Recurrent urinary tract infections \| \| K190z00 \| Urinary tract infection, site not specified NOS \| \| K190500 \| Urinary tract infection \| \| K190400 \| Chronic urinary tract infection \| \| K190.00 \| Urinary tract infection, site not specified \| \| K15..00 \| Cystitis \| \| K150.00 \| Acute cystitis \| \| 14D4.00 \| H/O: recurrent cystitis \| \| K155.00 \| Recurrent cystitis \| \| K15z.00 \| Cystitis NOS \| \| K152z00 \| Other chronic cystitis NOS \| \| K152y00 \| Chronic cystitis unspecified \| \| K15y.00 \| Other specified cystitis \| \| K15yz00 \| Other cystitis NOS \| \| 1J4..00 \| suspected UTI \| |
| --- | --- | --- | --- | --- | --- | --- | --- | --- | --- | --- | --- | --- | --- | --- | --- | --- | --- | --- | --- | --- | --- | --- | --- | --- | --- | --- | --- | --- | --- | --- | --- | --- | --- | --- | --- | --- |
|  |
| \| 1A53.11 \| C/O - loin pain \| \| --- \| --- \| \| R090C00 \| Loin pain \| \| 1A55.00 \| Dysuria \| \| 1A...12 \| Urinary symptoms \| \| K197.00 \| Haematuria \| \| 1A1..11 \| Frequency of micturition \| \| 1979 \| Suprapubic pain \| \| 1A12.00 \| Frequency of micturition \| \| 1AZ6.00 \| Lower urinary tract symptoms \| |

**Code list 2: Read code suggesting urine sent for culture**

| **Read code** | **Read term** |
| --- | --- |
| 4JJ..12 | Mid-stream urine sample |
| 4JJ..12 | Mid-stream urine sample |
| 461..11 | MSU - general |
| 4JJ2.00 | MSU sent for bacteriology |
| 4JJ2.00 | MSU sent for bacteriology |
| 4JJ1.00 | MSU sent for C/S |
| 4JJ1.00 | MSU sent for C/S |
| 4615 | MSU sent to lab. |
| 4615 | MSU sent to lab. |
| 46U..00 | Urine culture |
| 46U..00 | Urine culture |
| 46U8.00 | Urine culture - Bacteria OS |
| 4JJ..13 | Urine for culture |
| 4JJ..13 | Urine for culture |
| 46f3.00 | Urine leucocyte test = ++ |
| 46f4.00 | Urine leucocyte test = +++ |
| 46D..00 | Urine microscopy - general |
| 46DZ.00 | Urine microscopy - general NOS |
| 46G..00 | Urine microscopy: cells |
| 46X0.00 | Urine nitrite positive |
| 4146 | Urine sample sent to Lab |
| 4JJ3.00 | Urine sent for culture |
| 4JJ3.00 | Urine sent for culture |

**Code list 3: ICD-10 codes indicating a hospital diagnosis of UTI**

N30.0 Acute cystitis

N30.9 Cystitis, unspecified

N39.0 Urinary tract infection, site not specified
