# Supplementary material for: Incidence and antibiotic prescribing for clinically diagnosed urinary tract infection in older adults in UK primary care, 2004-2014
Source: PLoS One. 2018 Jan 5;13(1):e0190521. doi: 10.1371/journal.pone.0190521 (PMC5755802; doi:10.1371/journal.pone.0190521)
Supplement: S1 Table — Every third month shown. (DOCX) [file pone.0190521.s003.docx]

**S1 Table. Incidence rates per 100 person years at risk for community acquired UTI 2004 – 2014. Every third month shown.**
