# Supplementary material for: Incidence and antibiotic prescribing for clinically diagnosed urinary tract infection in older adults in UK primary care, 2004-2014
Source: PLoS One. 2018 Jan 5;13(1):e0190521. doi: 10.1371/journal.pone.0190521 (PMC5755802; doi:10.1371/journal.pone.0190521)
Supplement: S2 Table — (DOCX) [file pone.0190521.s004.docx]

**S2 Table. Mean age (years) for each age group for each study year**

|  |  | **MEAN AGE (YEARS) FOR EACH AGE-GROUP FOR EACH STUDY YEAR** | | | | | | | | | | |
| --- | --- | --- | --- | --- | --- | --- | --- | --- | --- | --- | --- | --- |
|  | **Age-group** | **2004** | **2005** | **2006** | **2007** | **2008** | **2009** | **2010** | **2011** | **2012** | **2013** | **2014** |
| **Men** | **65-74** | 69.2 | 69.2 | 69.2 | 69.2 | 69.2 | 69.1 | 69.1 | 69.0 | 69.0 | 69.0 | 69.0 |
|  | **75-84** | 79.0 | 79.0 | 79.0 | 79.0 | 79.0 | 79.0 | 79.0 | 79.0 | 79.0 | 79.0 | 79.0 |
|  | **85+** | 89.0 | 88.3 | 88.2 | 88.2 | 88.3 | 88.3 | 88.4 | 88.4 | 89.0 | 89.0 | 89.0 |
| **Women** | **65-74** | 69.3 | 69.4 | 69.4 | 69.3 | 69.3 | 69.2 | 69.2 | 69.1 | 69.0 | 69.0 | 69.0 |
|  | **75-84** | 79.3 | 79.2 | 79.2 | 79.2 | 79.2 | 79.2 | 79.2 | 79.2 | 79.2 | 79.2 | 79.2 |
|  | **85+** | 89.4 | 89.1 | 89.0 | 89.0 | 89.1 | 89.1 | 89.2 | 89.3 | 89.3 | 89.4 | 89.4 |
